# Thank Martin Luther that ciprofloxacin could cure your gonorrhoea? Ecological association between Protestantism and antimicrobial consumption in 30 European countries

**DOI:** 10.12688/f1000research.26709.1

**Published:** 2020-10-06

**Authors:** Chris Kenyon, Geoffrey Fatti

**Affiliations:** 1 HIV/STI Unit, Institute of Tropical Medicine, Antwerp, Belgium; 2 Division of Infectious Diseases and HIV Medicine, University of Cape Town, Cape Town, South Africa; 3 Division of Epidemiology and Biostatistics, Department of Global Health, Faculty of Medicine and Health Sciences, Stellenbosch University, Cape Town, South Africa

**Keywords:** antibiotic consumption, antimicrobial resistance, culture, Hofstede model, Protestant, Catholic, spandrel

## Abstract

**Background: **Higher consumption of antimicrobials plays an important role in driving the higher prevalence of antimicrobial resistance in Southern compared to Northern Europe. Poor controls on corruption (CoC), high uncertainty avoidance (UA) and performance vs. cooperation orientation (POCO) of societies have been found to explain much of this higher consumption in Southern European countries.  We hypothesized that these predictors were in turn influenced by the Protestant Reformation in the 16 ^th^ century onwards.

**Methods: **We used structural equation modelling (SEM) to assess the relationships between country-level proportions being Protestant, CoC, UA, POCO and four markers of antimicrobial consumption in the community (all antibacterials, cephalosporin, macrolides and fluoroquinolones).

**Results: **The proportion of a country that was Protestant was negatively correlated with the consumption of all antibacterials. SEM revealed that UA predicted all antibacterial consumption (direct effect coef. 0.15, 95% Confidence Interval [CI] 0.04-0.26). The proportion Protestant exerted an indirect effect on consumption (coef. -0.13, 95% CI -0.21- -0.05). This effect was mediated predominantly via its effect on UA (direct effect coef. 0.15, 95% CI 0.04-0.26). The model explained 37% of the variation in consumption.  Similar results were obtained for each of the other three classes of antimicrobials investigated.

**Conclusions: **Our results are compatible with the theory that contemporary differences in antimicrobial consumption in Europe stem in part from cultural differences that emerged in the Reformation. These findings may explain the differential efficacy of similar antibiotic stewardship campaigns in Northern and Southern European populations.

## Introduction

Countries in Southern Europe have been noted for some time to have a higher prevalence of antimicrobial resistance (AMR) than Northern European countries ^1, 2^. As an example, the prevalence of *Neisseria gonorrhoeae* resistance to ciprofloxacin varies over three fold between countries in Europe ^3^. 

The major determinant of these variations in AMR is the higher consumption of antimicrobials (AMC) in Southern European countries ^3, 4^. Fluoroquinolone consumption for example varies 6-fold between European countries and is associated with the prevalence of ciprofloxacin resistance in *N. gonorrhoeae* ^3^. What is less clear is what the underlying reason is for the variations in AMC ^4, 5^.

Previous studies have found a range of cultural and structural factors underpin the large variations in the consumption of fluoroquinolone and other antibiotics between European countries and globally ^5– 10^. The act of prescribing an antimicrobial is highly social. Providing an antimicrobial represents the doctor’s concern for the patient, legitimizes the patient’s sick-role and reinforces the doctor’s claim to expert knowledge ^8, 10– 13^. These vary between countries as do patients perceptions of the need for antimicrobials to treat infections ^8, 11, 13, 14^. One study compared a Dutch and Belgian city, 60km apart, both Dutch speaking but the two cities being historically Protestant and Catholic, respectively ^14^. Where the Dutch labelled their upper respiratory tract infections (URTI) as ‘colds’ or ‘flu’ the Belgians labelled most episodes as ‘bronchitis’ and used more antimicrobials. In general, the researchers found that those from a Protestant background were more sceptical about using antimicrobials than those from Catholic backgrounds. This type of observation has led to some authors to speculate that there may be a negative association between the proportion of a country that is Protestant and antimicrobial consumption ^2, 12, 15^. Others have contested this claim ^11^, but no one has, to the best of our knowledge, formally tested this association. In this paper, we test the hypothesis that the proportion of a country’s population that are reported to be Protestant is negatively associated with AMC in Europe. Furthermore, we assess if the pathway through which this operates is via cultural and structural factors associated with Protestantism and AMC.

Two cultural dimensions have consistently been found to explain variations in AMC both within Europe and worldwide ^5– 8, 13, 14, 16^. These are Hofstede’s uncertainty avoidance index (UA) and performance-orientation versus cooperation-orientation index (POCO) ^5– 8, 13, 14, 16^. Both indices are negatively associated with both the proportion of a country that is Protestant ^17^ and AMC ^5– 8, 16^. The UA index is a measure of the extent to which a society feels threatened by ambiguous or unknown situations. In high UA cultures, individuals feel discomfort and stress in unstructured situations that are novel ^15^. The POCO index (also termed the masculinity index) provides a measure of how performance-oriented cultures are. In high POCO cultures ego needs, assertiveness, targets and success are emphasized, whereas cooperation-oriented cultures place more focus on caring for all members of society, including the weak ^14^. An important reason not to take an antibiotic for an illness such as an URTI is that this will select for AMR – an adverse effect for the population at large. It has been argued that this low-POCO populations are more receptive to this population-benefit message than high POCO-populations ^8, 18^. 

The key structural factor found to be associated with AMC is control of corruption (CoC) at country and regional levels ^19, 20^. Countries with high levels of corruption (low CoC) have poorer institutional controls on prescribing practices and greater influence of pharmaceutical companies both of which can result in increased AMC ^12, 19, 20^. Previous ecological studies have found associations between national level CoC, UA, POCO and the proportion of the country being Protestant ^7, 8, 13, 16, 21^. Multivariate country-level studies from within Europe and elsewhere have also found that UA and POCO ^7, 8, 18, 22^ are predictors of AMC and an additional study has found that UA, POCO and CoC are independently associated with AMC ^16^. In this ecological study we use structural equation modelling to assess the association between countries’ population proportions being protestant and AMC, modelling UA, POCO and CoC as potential mediating variables. 

## Methods

### Data

***Antibiotic consumption.*** Data from the European Surveillance of Antimicrobial Consumption (ESAC) were used as a measure of national general population-level antimicrobial drug consumption ^23, 24^. ESAC reports antimicrobial consumption as the number of defined daily doses per 1000 inhabitants (DID) following the World Health Organization guidelines ^25^. In our study, we used four measures of country-specific antimicrobial drug use in ambulatory care: Total antibacterials for systemic use (ATC group J01), Cephalosporins/other Beta lactams (ATC group J01D), fluoroquinolones (ATC group J01MA), macrolides, lincosamides and streptogramins (ATC group J01F). Data was available from 1998 to 2018 and we used this data to calculate the peak consumption of each of these four classes of antimicrobial over this time period. All countries with available data were used in all the analyses. This data is available from ESAC without restrictions: https://www.ecdc.europa.eu/en/antimicrobial-consumption/database/quality-indicators

***Percent protestant.*** The proportion of a national population that was protestant was sourced from the Pew Research Centre estimates for 2010: https://www.pewforum.org/2011/12/19/table-christian-population-as-percentages-of-total-population-by-country/

***UA and POCO.*** Individual scores for UA and POCO were obtained for each country from Hofstede Insights, freely available from ( https://www.hofstede-insights.com/product/compare-countries/) is denoted as masculinity on the website.

***Control of corruption.*** The World Bank has provided indicators pertaining to six dimensions of governance since 1996. We used the dimension (control of corruption) that has been found to be most closely linked to AMC ^12, 16, 17^: Control of corruption (CoC) is defined as the country-level extent to which public power is exercised for private gain, including both petty and grand forms of corruption, as well as the capture of the state by elites. The index provides each country's score in units of standard normal distribution, ranging from approximately -2.5 (low CoC) to 2.5 (high CoC). The values used are average scores for the years 2013 to 2015, which we calculated from the original data, which was obtained from the following site: http://datatopics.worldbank.org/world-development-indicators/.

### Data analysis

A correlation matrix was performed to investigate the relationship between the different variables hypothesized to be associated with AMC. This approach was complemented by scatterplots of the associations between percent Protestant and AMC. We used structural equation modelling (SEM) to analyse the factors predicting AMC. SEM provided a way to analyse and graphically represent the complex direct and indirect pathways between endogenous and exogenous variables. All variables were assessed for non-linearity. No transformation was necessary. The analyses were performed using the SEM-builder in STATA 16. A P-value of less than 0.05 was used as the threshold of statistical significance.

## Results

Complete data was available for 29 of 30 countries with AMC data in the ESAC database ( Table 1). Data for UA and POCO were missing for Cyprus. Peak total antibacterial consumption varied fourfold between 10.1 DID in the Netherlands and 40.4 DID in Greece (median 21.2 [IQR 16.7-23.9]; Table 2).

**Table 1.** National antimicrobial consumption (defined daily doses/1000 individuals/day), Uncertainty Avoidance (UA) Index, Power oriented vs. cooperation oriented (POCO) Index, Percent Protestant in 30 European countries.

Country	Protestant (%)	UA	POCO	CoC	All AB	Fluoroquinolone	Macrolide	Cephalosporin
**Austria**	5.1	70	79	1.51	14.2	1.5	3.9	1.95
**Belgium**	1.4	94	54	1.61	23.9	2.77	3.78	4.41
**Bulgaria**	0.6	85	40	-0.26	22.7	2.87	3.97	4.45
**Croatia**	0.3	80	40	0.2	21.1	1.61	3.5	4.11
**Cyprus**	0.1	NA	NA	1.11	29.2	7.02	4.0	7.01
**Czech Republic**	3.5	74	57	0.34	17.4	1.37	3.98	2.21
**Denmark**	81.9	23	16	2.29	16.7	0.57	2.66	0.05
**Estonia**	21.2	60	30	1.26	14.4	0.92	2.49	1.22
**Finland**	80.2	59	26	2.21	18.6	0.95	2.38	2.38
**France**	1.8	86	43	1.32	28.8	2.18	6.06	4.82
**Germany**	34.8	65	66	1.83	14.5	1.51	2.8	3.22
**Greece**	0.3	112	57	-0.08	40.4	2.97	12	9.5
**Hungary**	21.6	82	88	0.21	21.1	2.69	4.14	3.35
**Iceland**	91.3	50	10	1.89	23.1	1.12	1.89	0.76
**Ireland**	5.1	35	68	1.59	21.3	1.04	4.38	2.01
**Italy**	1.3	75	70	0.01	23.7	3.46	5.3	3.87
**Latvia**	20.1	63	9	0.4	12.1	1.07	2.04	0.66
**Lithuania**	1.4	65	19	0.53	22.1	1.41	2.38	3.2
**Luxembourg**	3.2	70	50	2.1	25.4	2.85	5.5	5.78
**Malta**	1.1	96	47	0.91	20.7	3.06	4.53	5.68
**Netherlands**	21.8	53	14	1.97	10.1	0.91	1.5	0.13
**Norway**	83.3	50	8	2.25	16.5	0.56	2.1	0.29
**Poland**	0.4	93	64	0.63	23.8	1.48	6.01	3.99
**Portugal**	1.6	99	31	0.95	22.4	3.7	4.4	3.77
**Romania**	6.3	90	42	-0.11	28	3.71	3.18	5.33
**Slovakia**	9.8	51	100	0.14	25.8	2.41	6.3	6.21
**Slovenia**	1.2	88	19	0.74	16.8	1.53	3.91	0.91
**Spain**	1	86	42	0.71	25.6	2.88	3.4	2.62
**Sweden**	64.4	29	5	2.23	15.5	1.09	1.02	0.62
**United Kingdom**	54.5	35	66	1.77	18.5	0.62	3.24	1.08

**Table 2.** Pearson’s correlation coefficients between study variables. Significance levels are highlighted by * for P<0.05, ** for P<0.005. Abbreviations: All AB – All antibacterials, CoC – Control of Corruption; UA- Uncertainty Avoidance Index; POCO – Performance Oriented vs. Cooperation Oriented society.

	Protestant (%)	UA	POCO	CoC	All AB	Cephalosporin	Macrolide	Fluoroquinolone
**Protestant (%)**	1.0000							
**UA**	-0.69 ^*^	1.00						
**POCO**	-0.46 ^*^	0.23	1.00					
**CoC**	0.67 ^**^	-0.62 ^**^	-0.37 ^*^	1.00				
**All AB**	-0.38 ^*^	0.55 ^**^	0.34	-0.44 ^*^	1.00			
**Cephalosporins**	-0.59 ^**^	0.68 ^**^	0.52 ^**^	-0.52 ^**^	0.85 ^**^	1.00		
**Macrolides**	-0.50 ^**^	0.57 ^**^	0.54 ^**^	-0.47 ^*^	0.78 ^**^	0.79 ^**^	1.00	
**Fluoroquinoloes**	-0.53 ^**^	0.74 ^**^	0.38 ^*^	-0.42 ^*^	0.62 ^**^	0.73 ^**^	0.38 ^*^	1.000

**Figure 1.**
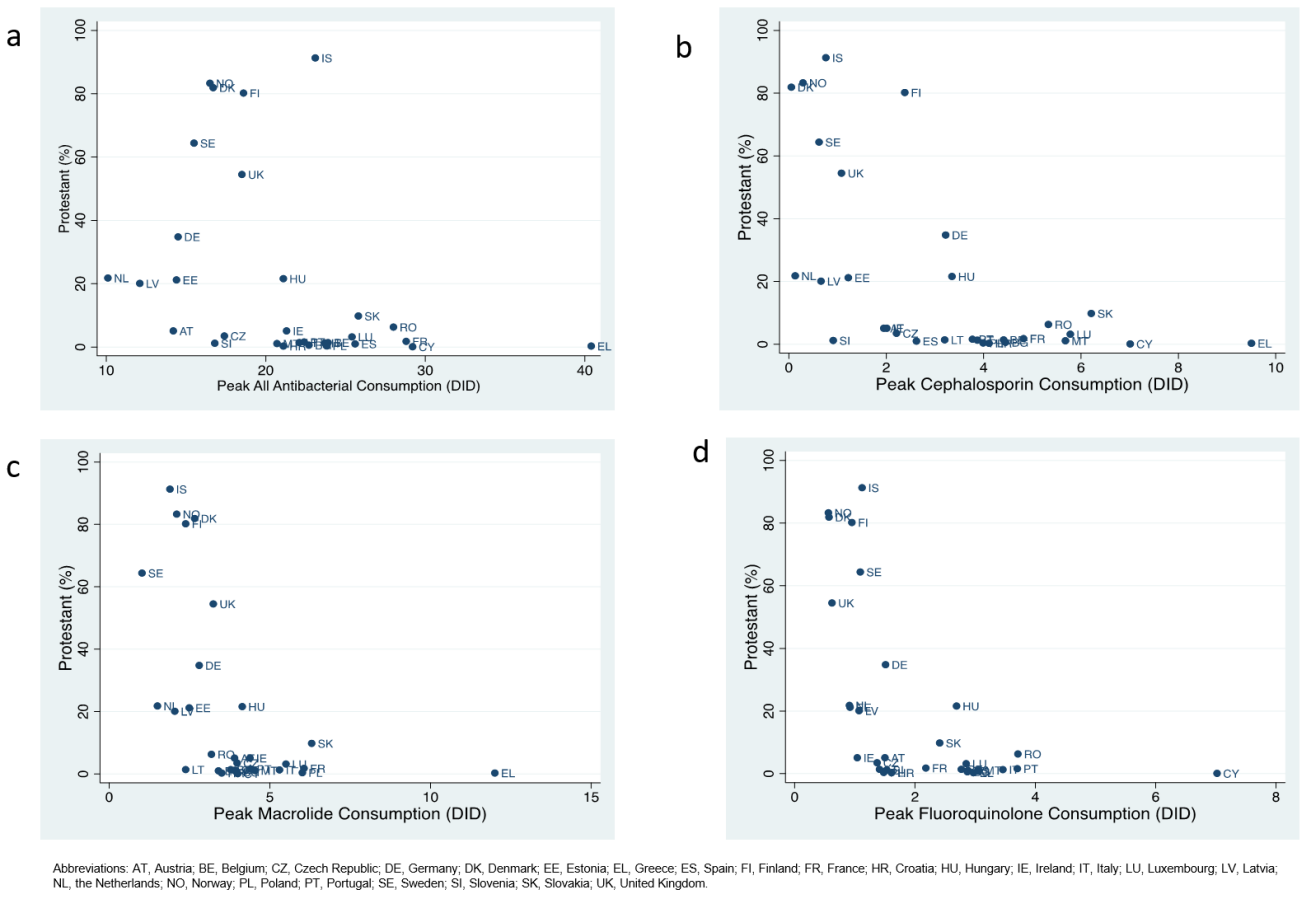
Scatter diagrams of percent of a country that is Protestant and consumption (defined daily doses/ 1000 individuals/ day – DID) of all antibacterials ( **a**), cephalosporins ( **b**), macrolides ( **c**) and fluoroquinolones ( **d**) in 30 European countries.

The proportion of a country that was Protestant was negatively correlated with the consumption of all antibacterials (r= -0.38; P=0.041), cephalosporins (r= -0.59; P=0.001), macrolides (r= -0.50; P=0.005) and fluoroquinolones (r= -0.53; P=0.003) as well as UA (r= -0.69; P<0.001) and POCO (r= -0.46; P=0.012). It was also positively associated with CoC (r= 0.67; P<0.001; Table 2; Figure 1). UA was positively correlated with all four categories of AMC (all r≥ 0.55; P<0.005) and negatively associated with CoC (r= -0.62; P<0.001). POCO was correlated with three of four categories of AMC and also negatively associated with CoC (r= -0.37; P=0.049). CoC was negatively associated with all four categories of AMC (all r < -0.43; P<0.05).

### Predictors of total antibacterial consumption

**Figure 2.**
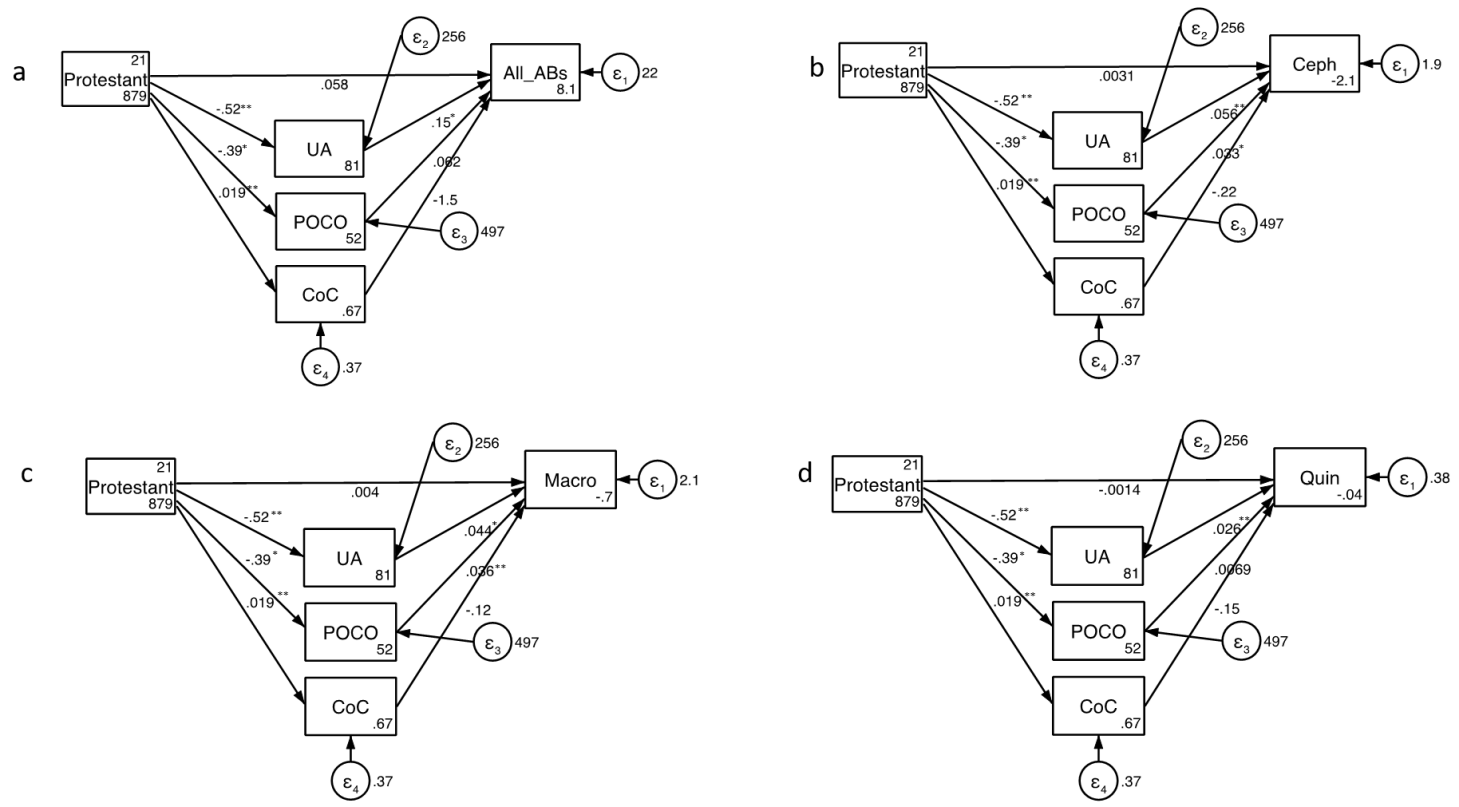
Structural equation modelling of predictors of consumption of total antibacterials ( **a**), cephalosporins ( **b**), macrolides ( **c**) and fluoroquinolones. The numbers next to the arrows are coefficients and their significance is indicated as follows: * P<0.05, ** P<0.005 (Abbreviations: All AB – All Antibacterial consumption; Ceph – Cephalosporins, CoC – Control of Corruption, Macro – Macrolides; POCO – Performance Oriented vs. Cooperation Oriented Index; Quin – Fluoroquinolones; UA - Uncertainty Avoidance Index).

Structural equation modelling revealed that UA predicted community antimicrobial consumption (direct effect coef. 0.15, 95% Confidence Interval [CI] 0.04-0.26; Figure 2; Extended Data). The percent Protestant exerted only an indirect effect on consumption (coef. -0.13, 95% CI -0.21- -0.05; Extended Data). This effect was mediated predominantly via its effect on UA (direct effect coef. 0.15, 95% CI 0.04-0.26). The model explained 37% of the variation in consumption (). 

### Analysis by antibiotic class

The SEM analysis found that the consumption of each class of antimicrobial was positively associated with UA and POCO ( Figure 2; Extended Data). Only in the case of POCO predicting fluoroquinolone consumption was this association not statistically significant ( Figure 2; Extended Data). Once again, the percent Protestant only exerted an indirect effect on AMC. This effect was mediated by UA and POCO both of which were negatively associated with percent Protestant. For each class of antimicrobial, the effect of UA explained the greatest proportion of variation in consumption (Extended Data). Overall the models explained 52% to 61% of the variation in consumption (Extended Data). 

## Discussion

Our results recapitulate those from other studies that UA and, to a lesser extent, POCO mediate a considerable proportion of the variation of AMC in Europe ^6, 8, 10, 16^. Whilst percent Protestant has little to no direct effect on AMC, our analysis found it has an indirect effect via its negative association with UA and POCO. The fact that this effect was similar for all 4 categories of antimicrobials investigated makes this finding more robust.

These results are compatible with the theory that the profound rupture in European society in the 16 ^th^ century induced by the Reformation may have had enduring effects that explain a portion of the contemporary variations in antimicrobial consumption between European countries. Our study provides evidence supportive of the thesis that this effect is mediated via Protestantism’s effect on two cultural variables – UA and POCO.

The reason that predominantly Catholic countries have higher UA and POCO scores is not clear, but may be related to factors such as the rituals and certainty-of-Faith that have characterized Catholicism ^11, 14, 17, 26^. It has been argued that Protestant teaching provided less certainty-of-Faith, encouraged more discussion, discouraged rituals, promoted austerity/simplicity and placed the locus of control less in the priest or church but in each individual ^12, 17, 26^. Protestant populations may therefore be more tolerant of uncertainty, have less faith in quick fix solutions and be more amenable to discussions about therapeutic strategies not involving antibiotics ^12, 17^. Protestants have also been found to have more trust in the ‘self-healing power of the body’, which has in turn been found to be correlated with scepticism towards the use of antibiotics ^14^. Both patients and doctors in historically Protestant, low-UA populations may therefore be more receptive to antibiotic stewardship messages that strongly discourage antibiotics for infections such as URTIs ^2^. High-UA societies on the other hand, may be less receptive to stewardship messages due to the uncertainty of ‘what if the URTI is caused by a bacterial infection?’ ^4, 8, 10^.

There are a number of important limitations to this analysis. We should be extremely guarded about drawing causal inferences concerning processes hundreds of years ago based on contemporary data from a small selection of countries. We did not control for possible confounders in the association between proportion of the population Protestant and AMC. We did not control for socio-economic markers such as GDP/capita as previous analyses have found that these did not explain differences in AMC within Europe ^16, 18^. Our sample size was also too small to justify controlling for a large range of confounders. There are a number of fundamental problems with classifying countries by religion. To an important extent, countries have a fluid mix of particular religions and both the relative sizes of the religions and the nature of these religions vary over time ^26^. There are also considerable differences within a religion such as differences between Catholicism in different countries and regions ^17^. This problem is compounded by our percent-Protestant-variable which combines a heterologous group of Catholic, Orthodox and other groups into one non-Protestant category. This classification could, however, be defended since our hypothesis is that low AMC was a byproduct (spandrel) of the Protestant Reformation. This line of thought is strengthened by a European study that found that the percent of the population describing themselves as atheist as opposed to religious was strongly associated with lower AMC ^9^. The study did not include a religious denomination variable but the authors noted evidence that secularization has been more pronounced in historically Protestant countries ^26^ and concluded that the lower AMC in these countries may be indirectly related to Protestantism. We considered reverse causation unlikely, but this cannot be excluded. Finally, the various dimensions of Hofstede’s model have been criticized as being over-simplifications of cultural differences ^27^.

A spandrel is an architectural term referring to the tapering triangular space formed by the intersection of two rounded arches at right angles ^28^. Gould argued that “evolutionary biology needs such an explicit term (spandrels) for features arising as by-products, rather than adaptations, whatever their subsequent exaptive utility…Causes of historical origin must always be separated from current utilities; their conflation has seriously hampered the evolutionary analysis of form in the history of life” ^28^. Previous analyses have found evidence of a range of spandrels exerting their effects hundreds of years later ^29, 30^. One example comes from Southern Africa, where differential HIV prevalence by ethnic group has been linked to distant historical processes. A number of colonial policies that were imposed on indigenous ethnic groups practicing polygamous partnering resulted in dense sexual networks that facilitated the spread of HIV in these groups hundreds of years later ^29, 31^. In contrast, Southern African ethnic groups from European origin have low sexual network connectivity and HIV prevalence. This low connectivity stems primarily from historical processes in Europe many centuries prior that resulted in forms of monogamous partnering being normative ^29^. Appreciating this historical connection has been shown to have three major benefits. Firstly, it provides an explanation as to how dramatic differences in behaviour and disease outcome can emerge. Secondly, it provides clues to the high HIV-prevalence populations as to how to tackle the underlying determinants of high prevalence. Thirdly, it does this in a non-judgemental way. Contemporary populations cannot be held responsible for events and processes occurring centuries prior ^29^.

Similar arguments could be made as to the relevance of the current analysis. It provides a possible deep historical explanation for how differences in AMC have emerged in Europe. It suggests that the lower AMC in predominantly Protestant countries could be explained by cultural differences that emerged in a process starting centuries ago. If this is correct, then this insight should generate greater understanding for how much harder antibiotic stewardship work is in non-Protestant countries. This is not an argument that stewardship is impossible or should not be attempted, but rather that campaigns might need to be more intense to achieve the same outcomes. It also provides further evidence that stewardship efforts need to be adapted to the local cultural context ^6, 7^. A concrete example of this would be to incorporate rapid diagnostic tests (that can remove uncertainty about bacterial infections) as a part of stewardship campaigns in high UA populations ^7^. If evidence were to come to light of ways to decrease uncertainty avoidance and favour cooperation- vs. performance-orientation these may also be considered as upstream interventions to reduce AMC. 

## Data availability

### Underlying data

All data underlying the results are available as part of the article and no additional source data are required.

### Extended data

Figshare: Thank Martin Luther that ciprofloxacin could cure your gonorrhoea? Ecological association between Protestantism and antimicrobial consumption in 30 European countries, https://doi.org/10.6084/m9.figshare.12994439.v1 ^32^.

This project contains the following extended data:

- SEM models and estimates of Goodness of Fit

Data are available under the terms of the Creative Commons Attribution 4.0 International license (CC-BY 4.0).
