# Supplementary material for: Thank Martin Luther that ciprofloxacin could cure your gonorrhoea? Ecological association between Protestantism and antimicrobial consumption in 30 European countries
Source: F1000Res. 2022 May 6;9:1200. Originally published 2020 Oct 6. [Version 2] doi: 10.12688/f1000research.26709.2 (PMC9114826; doi:10.12688/f1000research.26709.2)
Supplement: Peer review history

# Peer review: Reviewer response for version 2

**DOI:** 10.5256/f1000research.133783.r128893

**Published:** 2022-05-17

**Authors:** Harvey Morgan Scott

**Affiliations:** 1 Department of Veterinary Pathobiology, College of Veterinary Medicine and Biomedical Sciences, Texas A&M University, College Station, TX, USA

I was intrigued by the title of this paper, as I suspect many other readers have been (or will be). In the back of my mind, I was asking: "Is this a tongue-in-cheek update of Emile Durkheim's (the 'father' of sociology) famous treatise on suicide, focusing now on antimicrobial consumption as a means to illustrate the concept of ecological fallacy?". In Durkheim's work (1897 ^1^; as interpreted by Morgenstern (1995) ^2^), an ecological analysis of suicide rates by region of then-Prussia in Europe showed a 7.6-fold increase in rates of suicide comparing the highest proportion Protestant versus the lowest proportion Protestant (blame (not thank) that on Martin Luther?). That was when analyzed in the aggregate, but showed only a 2-fold increase in suicide rates when analyzed at the individual-level by Durkheim himself. Still increased, but greatly upwardly biased estimates for individuals based on their religious affiliations. Of course, the present paper on AMC appears to lack the data granularity to confirm or refute the religion of those individuals either prescribing or else receiving antibiotics. And this isn't necessary, especially given the limitations and caveats that Kenyon and Fatti have provided. The authors have been careful to describe their analyses and conclusion as being entirely ecological, and we can accept this. Studies of the aggregate are indeed crucially important for exploring differences in the aggregate, especially when allocating resources or seeking solutions to complex social problems. However the social aggregations and normative consequences at the societal level, it is clear that in some way the aggregation is something less or more than the sum of parts (now moving into atomistic fallacy...). There are countries within the dataset where there are substantive regional north/south differences in the distribution of Protestant versus 'other' (Catholic, generally) and so both Germany and The Netherlands might have warranted some discussion to this effect. If Prussia still existed, it would be a great microcosm in which to explore the phenomena in the current paper! In other words, if the phenomenon is to be believed, then there should be somewhat obvious differences in prescribing rates between Hamburg versus Munich (as one example). Likely insufficient for statistical analysis, but would serve as a nice confirmation or refutation vignette. In addition, Cyprus as a country poses more problems than simply missing data on UA and POCO; that is, since it is divided into a Turkish (largely Muslim) north and Greek (largely Orthodox Christian) south and complex governance and conflict issues likely pose problems in interpretation beyond those listed in the paper. And, it is no doubt an obvious caveat that a tourist from a Scandinavian country holidaying at a Mediterranean beach resort should probably not wait until returning home before receiving an antibiotic for a newly acquired case of gonorrhea.

 I recognize the authors have added substantive discussion on the limitations of inference and I concur with those (e.g., temperature gradients, lack of control of other confounders, etc). It would be useful to also include reference to publicly available rates of bacterial infectious diseases per EU surveillance (or, at least summarize whether or not increased rates of AMC are related to reported disease (perhaps inaccurately).

 In summary, I do think a nod to the most famous of historical papers that explored relations between a significant pubic health outcome (suicide) and religion is warranted, especially since that work is commonly used as an exemplar of the 'Ecological Fallacy', at least among epidemiologists. The authors have invoked the religious origins of a potent social upheaval/reformation as leading to differences in prescribing and use of a modern technology hundreds of years later. A brief stop in the 19th century to acknowledge the work of a French sociologist who made similar conclusions seems warranted.

 One minor note:

 I was looking for IS in Figure 1 legend but couldn't find it. I imagine it is for Iceland based on Table 1 and % Protestant?

Is the work clearly and accurately presented and does it cite the current literature?

Yes

If applicable, is the statistical analysis and its interpretation appropriate?

Partly

Are all the source data underlying the results available to ensure full reproducibility?

Yes

Is the study design appropriate and is the work technically sound?

Partly

Are the conclusions drawn adequately supported by the results?

Partly

Are sufficient details of methods and analysis provided to allow replication by others?

Yes

Reviewer Expertise:

Antimicrobial resistance research, epidemiology

I confirm that I have read this submission and believe that I have an appropriate level of expertise to confirm that it is of an acceptable scientific standard, however I have significant reservations, as outlined above.

## References

[1] : Suicide (1897). *The Free Press reprint 1997* .

[2] : Ecologic studies in epidemiology: concepts, principles, and methods. *Annu Rev Public Health* .1995;16: 10.1146/annurev.pu.16.050195.000425 61-81 10.1146/annurev.pu.16.050195.000425 7639884

# Peer review: Reviewer response for version 1

**DOI:** 10.5256/f1000research.29490.r76564

**Published:** 2022-05-04

**Authors:** Peter J Collignon

**Affiliations:** 1 Infectious Diseases and Microbiology, Canberra Hospital, Australian National University, Acton, Australia

This is an interesting paper with the thesis being that a Catholic tradition will result in higher use of antimicrobials than having a protestant background. While there appears to be an association using the limited parameters looked at in this study, that is very different from being able to infer any cause-and-effect relationship, however. 

 The countries looked at that are “protestant” are generally colder as well. So maybe temperature has as big an effect. Or do those with a catholic tradition have more sexual encounters than those of a protestant background – especially as the focus is on *gonococcus*? But these and many other parameters were not measured to see what influence, if any, they might have in their correlation analysis.

 They only looked at a few parameters to assess the relationship with Antimicrobial consumption; poor controls on corruption (CoC), high uncertainty avoidance (UA) and performance vs. cooperation orientation (POCO) and proportion of a population that was protestant. These are not enough parameters given the complexities involved with antimicrobial resistance and consumption (e.g. many more parameters were looked at by Collignon P, Beggs JJ, Walsh TR, Gandra S, Laxminarayan R, in Anthropological and socioeconomic factors contributing to global antimicrobial resistance: a univariate and multivariable analysis. Lancet Planet Health. 2018 https://www.sciencedirect.com/science/article/pii/S2542519618301864?via%3Dihub) ^1^

 It is important to note that even of the limited parameters they looked at, the percent Protestantism, was the least associated parameter with antimicrobial use.  As they note “Once again, the percent Protestant only exerted an indirect effect on AMC. This effect was mediated by UA and POCO both of which were negatively associated with percent Protestant. For each class of antimicrobial, the effect of UA explained the greatest proportion of variation in consumption.”

 In a paper entitled “Corruption and use of antibiotics in regions of Europe” (their reference 19), Rönnerstrand, and Lapuente show that within countries in Europe, there are marked variations by regions and presumably not likely influenced by the percent of that country’s population that were protestant, as it also occurs within countries where there is little Protestantism e.g., Italy. What did correlate, however, were the local levels of corruption. This again is what these authors have shown in their data.

 So, in summary, the authors (Kenyon and Fatti) did show an association with Protestantism and antimicrobial consumption, but even though they only looked at limited parameters, their own data suggest religion itself plays little part in any cause and effect. It appears that lower consumption of antimicrobials is likely very much more associated with regions that have better governance and lower corruption rates, rate than their conclusion of cultural differences that emerged in the Reformation.

Is the work clearly and accurately presented and does it cite the current literature?

Partly

If applicable, is the statistical analysis and its interpretation appropriate?

Partly

Are all the source data underlying the results available to ensure full reproducibility?

Yes

Is the study design appropriate and is the work technically sound?

Partly

Are the conclusions drawn adequately supported by the results?

Partly

Are sufficient details of methods and analysis provided to allow replication by others?

Yes

Reviewer Expertise:

Microbiology, Infectious diseases, Antimicrobial resistance

I confirm that I have read this submission and believe that I have an appropriate level of expertise to confirm that it is of an acceptable scientific standard, however I have significant reservations, as outlined above.

## References

[1] : Anthropological and socioeconomic factors contributing to global antimicrobial resistance: a univariate and multivariable analysis. *The Lancet Planetary Health* .2018;2(9) : 10.1016/S2542-5196(18)30186-4 e398-e405 10.1016/S2542-5196(18)30186-4 30177008

# Response

**Published:** 2022-05-04

**Authors:** Chris Kenyon

**Affiliations:** Institute of Tropical Medicine, Antwerp, Belgium

We thank the reviewer for their useful comments. We agree that we need to be very careful with the conclusions we draw from this study for all the reasons outlined by the reviewer. We acknowledge that the Collignon *et al*., paper referred to by the reviewer is one of the most definitive ecological analyses of the drivers of AMR globally. This is however different to our research questions which were: what are the determinants of antimicrobial consumption (AMC), is the percent of a countries population that is Protestant associated with AMC and if so is this association a direct or an indirect one (via UA, POCO or CoC)? 

 As the reviewer notes, we find that there is a weak positive association and the effect is indirect- acting via UA and POCO. One parsimonious explanation for these findings is that an upstream determinant of UA and POCO is the percent of the population that is Protestant. As we note in the discussion, while we cannot prove that this is the causal pathway, this is at least a plausible way to interpret the data. To the best of our knowledge, this is the first time that statistical evidence has been produced to back up the percent Protestant-POCO/UA-AMC pathway. 

 Once again we have endeavoured in our discussion to make it very clear that whilst we find some statistical support for this pathway, this does not constitute strong evidence that this pathway played any role in the genesis of differential levels of AMC in European countries.

 To address the reviewer's valid concerns, we have added the Collignon *et al.* reference and the following text in the discussion (4th paragraph):

*We also did not control for differences in environmental temperatures. Southern European countries tend to be both hotter and more Catholic than Northern European countries. A previous ecological study has found that environmental temperature is associated with the prevalence of certain types of antimicrobial resistance ^33^*

# Peer review: Reviewer response for version 1

**DOI:** 10.5256/f1000research.29490.r79088

**Published:** 2021-03-03

**Authors:** Christoph M. Tang

**Affiliations:** 1 Sir William Dunn School of Pathology, University of Oxford, Oxford, UK

This is an interesting and somewhat provocative paper that shows a relationship between events several hundred years ago and current use of antimicrobials.

 The authors show a link between the consumption of antimicrobials in a given country and the prevalence of Protestantism.

 As the authors correctly acknowledge in their discussion, studies of this nature cannot derive causal relationships (and only highlight associations) and they acknowledge that many potential confounding factors are likely to be at play. They did not control for socio-economic markers (eg GDP), reasoning that ' *these did not explain differences in AMC within Europe*'. Whilst this might be true when considering socioeconomic factors on their own, it does not mean that these factors would not impact findings when incorporated into a multivariate analysis. The authors also discuss the multiplicity of faiths within many individual countries (which report overall antibiotic consumption levels and not levels according to religion) and how the practice and teaching of the same religion can differ between countries ( *There are also considerable differences within a religion such as differences between Catholicism in different countries and regions*).

Is the work clearly and accurately presented and does it cite the current literature?

Yes

If applicable, is the statistical analysis and its interpretation appropriate?

Yes

Are all the source data underlying the results available to ensure full reproducibility?

Yes

Is the study design appropriate and is the work technically sound?

Partly

Are the conclusions drawn adequately supported by the results?

Yes

Are sufficient details of methods and analysis provided to allow replication by others?

Yes

Reviewer Expertise:

Microbial pathogenesis and virulence

I confirm that I have read this submission and believe that I have an appropriate level of expertise to confirm that it is of an acceptable scientific standard.
